# Does primary tumor location impact the prognosis of colorectal liver metastases patients after microwave ablation? - Lessons from 10 years’ experience

**DOI:** 10.18632/oncotarget.18764

**Published:** 2017-06-28

**Authors:** Fubo Zhou, Xiaoling Yu, Ping Liang, Zhiyu Han, Zhigang Cheng, Jie Yu, Fangyi Liu, Yi Hu

**Affiliations:** ^1^ Department of Interventional Ultrasound, Chinese PLA General Hospital, Beijing 100853, China; ^2^ Department of Oncology, Chinese PLA General Hospital, Beijing 100853, China

**Keywords:** colorectal liver metastases, microwave ablation, primary tumor location, prognosis, survival

## Abstract

Thermal ablation has been considered as an alternative for local curative intent in patients with unresectable colorectal liver metastases. The influence of primary tumor location on the prognosis of colorectal liver metastases patients who have undergone microwave ablation has yet to be determined. We reviewed 295 patients who underwent microwave ablation for colorectal liver metastases at our institution between March 2006 and March 2016. Univariate and multivariate analyses were performed to identify predictors of overall and progression-free survival. Technical success was achieved in 96.6% of patients (*n* = 289), with a post-procedural complication rate of 2.0% (*n* = 6). After a median follow-up of 24 (range, 2–86) months, comparable overall survival rates (*p* = 0.583) were observed in patients with different primary tumor locations. Patients with colorectal liver metastases originating from left-sided primary colon cancer exhibited a better progression-free survival than patients whose colorectal liver metastases had originated from right-sided primary colon cancer (hazard ratio: 0.67, 95.0% confidence interval: 0.48–0.94; *p* = 0.012), which was further confirmed in a multivariate analysis after adjustment for other potential prognostic factors. Stratification based on primary tumor location should be taken into consideration in the assessment of disease progression in patients who intend to undergo microwave ablation for colorectal liver metastases.

## INTRODUCTION

Colorectal cancer (CRC) is the third most common cancer worldwide, with an incidence of approximately 1.4 million cases per annum [1]. Disparities between left- and right-sided CRCs have long been investigated, including the epidemiological distribution, clinicopathological characteristics, molecular features, recurrence patterns, and survival outcomes [2–6]. In metastatic CRC (mCRC), the prognosis of patients with different primary tumor locations has also been under investigation. Price *et al*. [7] enrolled 2,972 mCRC patients and reported that patients with left-sided primary colon cancer had a better prognosis than patients with right-sided primary colon cancer [7]. In addition, a subgroup analysis of 1,738 patients from the same cohort [7] who received active therapy (chemotherapy with or without mastectomy) also revealed that patients with left-sided primary colon cancer had a superior survival outcome. Loupakis *et al.* [8] also demonstrated that patients with metastases arising from left-sided primary CRC (*n* = 2,027) had a better survival outcome than those patients with metastases arising from right-sided primary CRC after first-line chemotherapy with or without biological agents. These findings are consistent with those of previously published reports [9, 10].

The liver represents the most common site of distant metastases in patients with CRC. Approximately 25.0% of patients present with synchronous liver metastases (LMs) at initial diagnosis and another 50.0% will develop LMs during their disease course [11]. Although surgical resection is the gold standard of treatment for colorectal LMs (CRLMs), only 8.0%–27.0% of patients are suitable candidates for hepatectomy [12]. For unresectable CRLMs, thermal ablation has been developed to serve as an alternative for local curative intent [13]. Of the different ablative treatment modalities, microwave ablation (MWA) has gained particular interest due to its non-reliance on electrical conductivity, char resistance, reduced heat-sink effect, and simultaneous administration of multiple antennas [14]. The safety and efficacy of MWA for liver malignancies have been confirmed in our previous studies [15, 16]. For CRLMs, the reported 4-year overall survival (OS) rate ranges from 35.2%–41.0% after MWA [11, 17–19]. To the best of our knowledge, the prognosis of patients with CRLMs after MWA according to primary tumor location remains poorly understood. Therefore, we aimed to investigate the prognosis of patients with CRLMs after MWA according to primary tumor location.

## RESULTS

### Clinicopathological characteristics

One hundred and thirty-six patients who failed to meet the inclusion criteria were excluded. In total, 323 patients who underwent ultrasound-guided percutaneous MWA for CRLMs between March 2006 and March 2016 were enrolled in this study. Of these, 295 patients had complete follow-up data and were ultimately included in our analyses. One hundred and ninety-two patients were male and 103 patients were female, with a median age of 59 (range, 30–86) years. The median number and maximum diameter of the LMs were 1 (range, 1–3) and 2.9 (range, 0.9–4.8) cm, respectively. The 295 patients underwent 415 MWA sessions for CRLMs, with the primary tumor located in the left-sided colon in 89 patients, the right-sided colon in 94 patients, and the rectum in 112 patients. Two hundred and sixty-two patients (88.8%) underwent pre-ablation systemic chemotherapy. Systemic chemotherapy regimens included: 5-fluorouracil, leucovorin, and irinotecan (*n* = 211 patients; 71.5%); 5-fluorouracil, leucovorin, and oxaliplatin (*n* = 188 patients; 63.7%); capecitabine and oxaliplatin (*n* = 156 patients; 52.9%); capecitabine and irinotecan (*n* = 91 patients; 30.8%); and others (*n* = 35 patients; 11.9%). Bevacizumab and cetuximab were administered in 27 (9.2%) and 17 (5.8%) patients, respectively. One hundred and sixty-five patients (55.9%) exhibited partial remission after pre-ablation systemic chemotherapy. No significant differences in tumor responses to pre-ablation systemic chemotherapy were detected between the groups (*p* = 0.657). Detailed clinicopathological characteristics of the enrolled patients, according to primary tumor location are summarized in Table 1.

**Table 1:** Demographic characteristics of the enrolled patients

Characteristics	Left-sided colon (*n* = 89)	Right-sided colon (*n* = 94)	Rectum (*n* = 112)	*p*
Age, median (years, range)	57 (30–86)	59 (39–83)	61 (30–82)	0.453
Sex (F/M)	62/27	58/36	72/40	0.515
KPS score, Median (range)	90 (80–90)	90 (80–90)	90 (70–90)	0.620
TNM stage (I/II *vs*. III/IV)	42 *vs*. 47	36 *vs*. 58	49 *vs*. 63	0.470
Node status (positive *vs*. negative)	24 *vs*. 65	31 *vs*. 63	29 *vs*. 83	0.496
Histologic grade				0.531
(Well/moderately *vs*. poorly differentiated)	61 *vs*. 28	57 *vs*. 37	73 *vs*. 39	
Preablation CEA (µg/L)	14.6	18.4	15.9	0.463
Median, range	(1.67–951.6)	(1.78–792.8)	(1.01–1703)	
Smoking (Never/smokers)	56/33	62/32	76/36	0.764
Alcohol consumption ≤ 15 g/day *vs*. > 15 g/day	62 *vs*. 27	72 *vs*. 22	81 *vs*. 31	0.565
CRLM characteristics				
Metachronous/synchronous	63/26	62/32	72/40	0.611
Number (Median, range)	2 (1–3)	1 (1–3)	1 (1–3)	0.493
Largest size (cm)	2.6 (0.9–4.8)	3.0 (1.1–4.6)	3.1 (1.6–4.8)	0.236
(Median, range)				
Distribution (Unilobar/bilobar)	65/24	63/31	72/40	0.411
Response to pre-ablation systemic chemotherapy (*n*, %)				0.657
PR	52	49	64	
SD	14	23	25	
PD	12	10	13	

Abbreviations: CEA, carcinoembryonic antigen; F, female; M, male; KPS, Karnofsky performance status; PD, progressive disease; PR, partial response; SD, stable disease; TNM, tumor-node-metastasis.

### Treatment parameters, complications, and side-effects

The mean ablation power and duration of MWA treatment for each metastatic lesion were 51.2 (range, 45–60) watts and 496.3 (range, 200–1,410) seconds, respectively. No significant differences were detected among the three groups in terms of ablation power, duration, MWA sessions, and the number of microwave antennas (Table 2).

**Table 2:** MWA treatment parameters

Treatment parameters	Left-sided colon (*n* = 89)	Right-sided colon (*n* = 94)	Rectum (*n* = 112)	*p*
Ablation power (W)	50.6	50.4	52.4	0.372
Mean (range)	(45–60)	(50–60)	(45–60)	
Ablation time (s)	516.2	492.5	483.7	0.659
Mean (range)	(200–1,410)	(240–1,040)	(240–1,080)	
Ablation session	1.3	1.4	1.5	0.265
Mean (range)	(1–3)	(1–3)	(1–3)	
No. of microwave antennas	1.6	1.8	1.7	0.764
Mean (range)	(1–2)	(1–2)	(1–2)	

Technical success was achieved in 96.6% of patients (*n* = 289). Post-procedural complications were reported in 6 patients (2.0%) who underwent thoracocentesis for pleural effusion. Side-effects included: a transient fever (*n* = 136 patients; 46.1%), abdominal pain (*n* = 63 patients; 21.4%), and nausea (*n* = 25 patients; 8.5%).

### Overall survival outcomes

**Figure 1:**
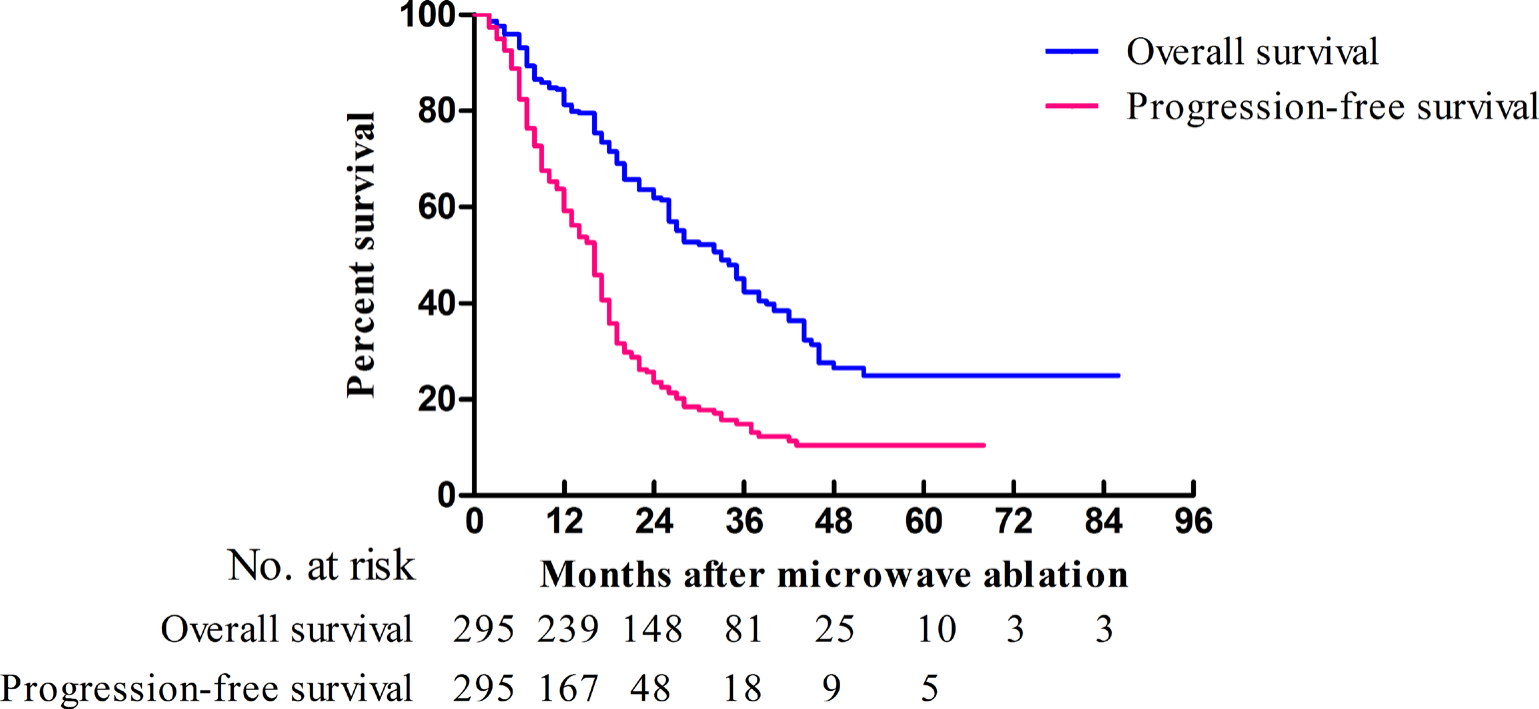
Kaplan-Meier curves of OS (blue) and PFS (red) in patients (*n* = 295) who underwent MWA for CRLMs

Patients were followed up for a median of 24 (range, 2–86) months, with a median OS of 33 (95.0% confidence interval [CI]: 28.5–37.5) months. The 1-, 3-, 5-, and 7-year OS rates were 81.3%, 42.3%, 24.9%, and 24.9%, respectively (Figure 1).

**Figure 2:**
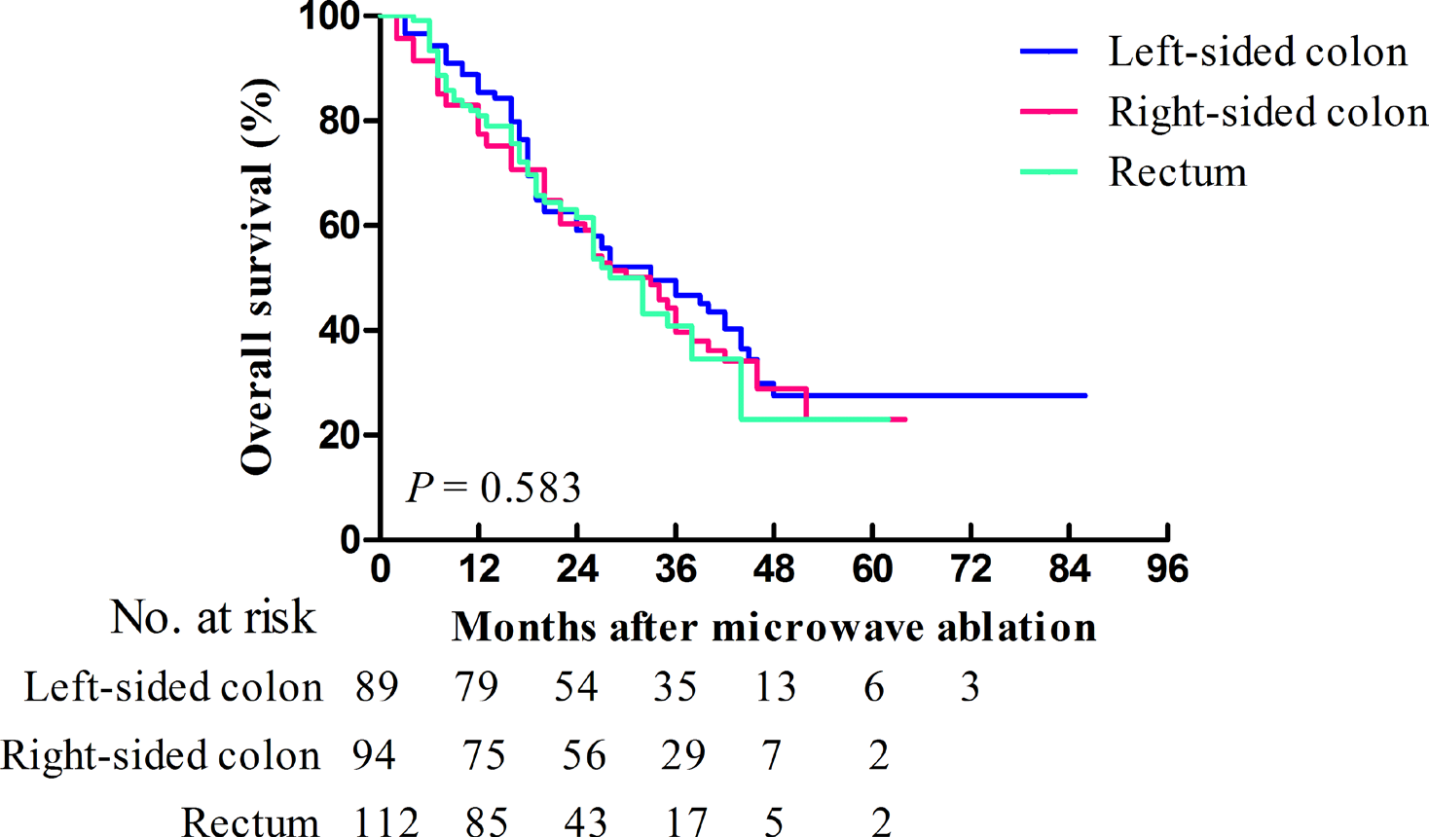
Kaplan-Meier curves of OS for patients (*n* = 295) who underwent MWA for CRLMs stratified according to primary tumor location Patients with primary tumors located in the left-sided colon, right-sided colon, and rectum are represented by the blue, red, and green lines, respectively.

The median OS times of patients with a primary tumor in the left-sided colon, the right-sided colon, and the rectum were 35 (95.0% CI: 24.9–45.1), 33 (95.0% CI: 25.8–40.2), and 32 (95.0% CI: 26.7–37.3) months, respectively (*p* = 0.583). The 1-, 3-, and 5-year OS rates were 85.4%, 46.7%, and 27.5%, 77.4%, 39.7%, and 23.1%, and 81.0%, 40.8%, and 23.0% for primary tumors located in the left-sided colon, the right-sided colon, and the rectum, respectively. No significant differences in OS rates were detected between groups with different primary tumor locations (left-sided colon vs. rectum, *p* = 0.356; left-sided colon vs. right-sided colon, *p* = 0.402; and rectum vs. right-sided colon, *p* = 0.909). The OS curves of patients with different primary tumor locations are presented in Figure 2.

From the univariate and multivariate analyses, the maximum diameter of the LMs and the patients’ responses to pre-ablation systemic chemotherapy were identified as prognostic factors for OS (Table 3). Patients with a maximum LM diameter of ≤ 3.0 cm were associated with a significantly better OS than patients with a maximum LM diameter of between 3.0 and 5.0 cm (hazard ratio [HR]: 0.36, 95.0% CI: 0.17–0.74; *p* < 0.01). Additionally, patients with stable or progressive disease after pre-ablation systemic chemotherapy were associated with a significantly poorer OS than patients with partial remission (HR: 1.87, 95.0% CI: 1.25–2.81 and HR: 2.36, 95.0% CI: 1.38–4.04, respectively; *p* < 0.01). In the multivariate analysis, the maximum diameter of the LMs, and the patients’ responses to pre-ablation systemic chemotherapy were confirmed as independent predictors of OS after adjustment for other potential prognostic factors (HR: 0.33, 95.0% CI: 0.14–0.78, *p* = 0.010; HR: 3.79, 95.0% CI: 2.46–5.82, *p* < 0.01).

**Table 3:** Univariate analyses of OS and PFS in patients who underwent MWA for CRLMs

Variable	OS		PFS	
	HR (95% CI)	*p*	HR (95% CI)	*p*
Age (years)	1.34 (0.79–2.26)	0.279	1.06 (0.69–1.62)	0.784
Sex	1.01 (0.61–1.69)	0.966	1.28 (0.84–1.97)	0.250
Primary tumor location		0.583		0.059
Rectum	1.02 (0.70–1.49)	0.909	0.85 (0.62–1.17)	0.359
Left-sided colon	0.85 (0.59–1.24)	0.402	0.67 (0.48–0.94)	0.012*
Right-sided colon	Reference	–	Reference	–
KPS score	1.05 (0.62–1.77)	0.855	1.03 (0.68–1.58)	0.876
TNM stage	0.67 (0.40–1.12)	0.123	1.27 (0.82–1.97)	0.294
Histological grade	1.09 (0.65–1.85)	0.742	0.93 (0.60–1.45)	0.760
Preablation CEA	1.06 (0.64–1.75)	0.816	1.32 (0.87–2.01)	0.194
Smoking status	0.75 (0.44–1.26)	0.272	1.06 (0.70–1.60)	0.783
Alcohol consumption	0.67 (0.38–1.17)	0.162	1.17 (0.77–1.78)	0.452
Characteristics of CRLM				
Timing	0.93 (0.51–1.72)	0.824	1.04 (0.63–1.71)	0.872
Number of CRLM	1.31 (0.80–2.17)	0.286	1.55 (1.03–2.31)	0.034*
Largest size (≤ 3 cm)	0.36 (0.17–0.74)	0.005*	0.51 (0.28–0.92)	0.026*
Distribution	1.32 (0.76–2.28)	0.323	1.63 (1.06–2.67)	0.324
Response to chemotherapy		< 0.01*		< 0.01*
PR	Reference	–	Reference	–
SD	1.87 (1.25–2.81)	< 0.01*	1.54 (1.03–2.30)	< 0.01*
PD	2.36 (1.38–4.04)	< 0.01*	2.79 (1.86–4.19)	< 0.01*

*p < 0.05.

Abbreviations: CEA, carcinoembryonic antigen; KPS, Karnofsky performance status; OS, overall survival; PD, progressive disease; PFS, progression-free survival; PR, partial response; SD, stable disease; TNM, tumor-node-metastasis.

### Progression-free survival outcomes

During follow-up, 214 patients (72.5%) exhibited disease progression, including local recurrence in 26 patients (8.8%), intrahepatic metastases in 106 patients (35.9%), and extrahepatic metastases in 82 patients (27.8%). The types of disease progression in each of the three groups are listed in Table 4. No significant differences were detected between patients with different primary tumor locations (*p* = 0.586).

**Table 4:** Type of disease progression after MWA according to primary tumor location

Type of disease progression	Left-sided colon (*n* = 89)	Right-sided colon (*n* = 94)	Rectum (*n* = 112)	*p*
Local recurrence, *n* (%)	11 (12.4)	7 (7.4)	8 (7.1)	0.586
Intrahepatic metastases, *n* (%)	31 (34.8)	33 (35.1)	42 (37.5)	
Extrahepatic metastases, *n* (%)	21 (23.6)	29 (30.9)	32 (28.6)	

Abbreviations: MWA: microwave ablation.

**Figure 3:**
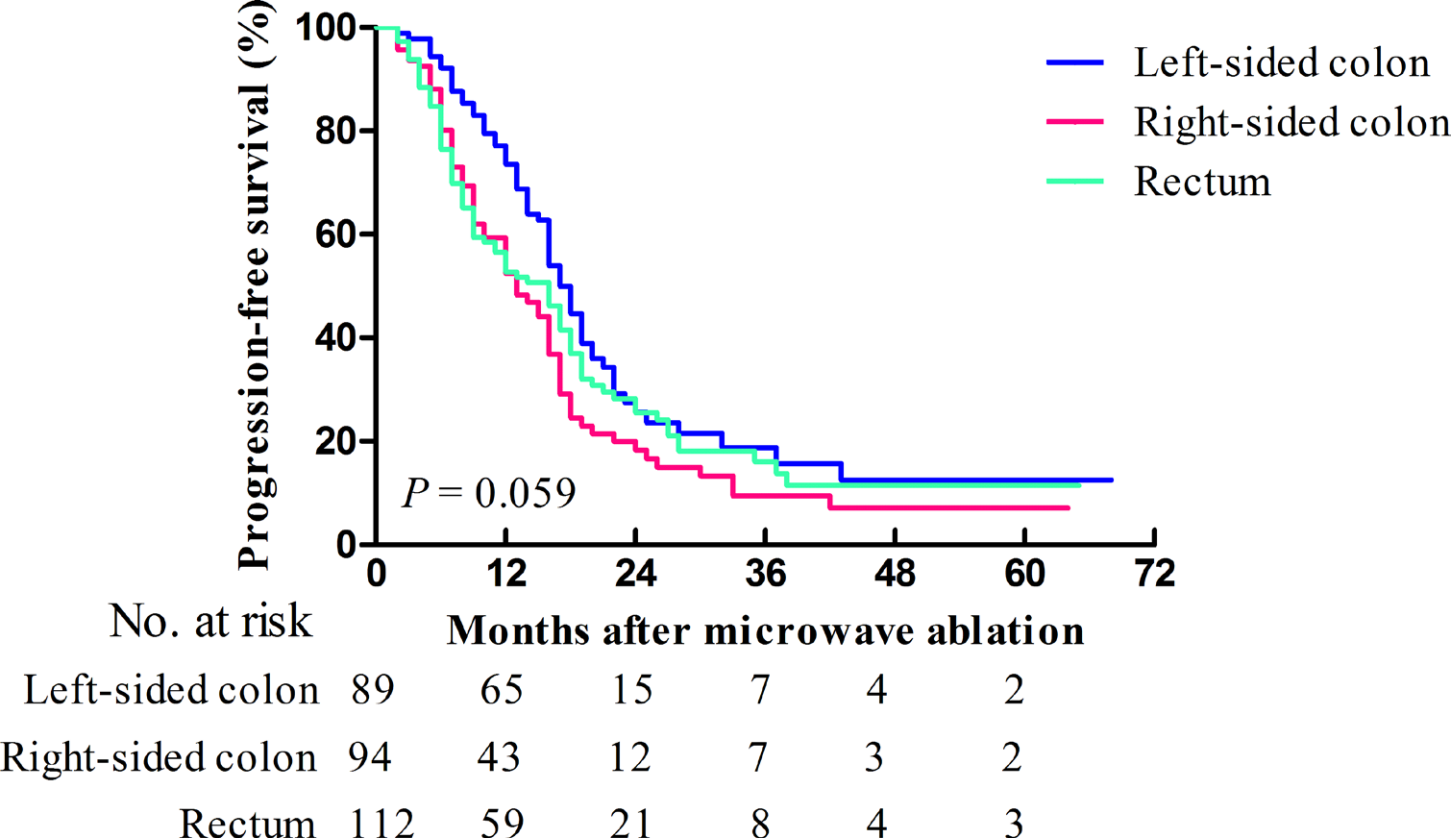
Kaplan-Meier curves of PFS for patients (*n* = 295) who underwent MWA for CRLMs stratified according to primary tumor location Patients with primary tumors located in the left-sided colon, right-sided colon, and rectum are represented by the blue, red, and green lines, respectively.

The median progression-free survival (PFS) was 16 (95.0% CI: 14.4–17.6) months, with 1-, 3-, and 5-year PFS rates of 59.2%, 14.9%, and 10.4%, respectively (Figure 1). The median PFS times of patients with a primary tumor in the left-sided colon, the right-sided colon, and the rectum were 17 (95.0% CI: 15.2–18.8), 13 (95.0% CI: 9.4–16.6), and 16 (95.0% CI: 12.1–19.9) months, respectively (*p* = 0.059). The PFS curves of patients with different primary tumor locations are presented in Figure 3. The 1-, 3-, and 5-year PFS rates were 73.5%, 18.8%, and 12.5%, 52.4%, 9.5%, and 7.1%, and 52.7%, 16.1%, and 11.5% for primary tumors located in the left-sided colon, the right-sided colon, and the rectum, respectively. The *p*-values between the groups with different primary tumor locations were as follows: 0.148 (left-sided colon vs. rectum), 0.012 (left-sided colon vs. right-sided colon), and 0.359 (rectum vs. right-sided colon). Patients with a primary tumor in the left-sided colon were associated with a significantly better PFS than patients with a primary tumor in the right-sided colon (HR: 0.67, 95.0% CI: 0.48–0.94; *p* = 0.012).

In the univariate analysis, primary tumor location, the number and maximum diameter of the LMs, and the patients’ responses to pre-ablation systemic chemotherapy were identified as independent predictors of PFS (Table 3). Patients with multiple LMs were associated with a significantly poorer PFS than patients with a solitary LM (HR: 1.55, 95.0% CI: 1.03–2.31; *p* = 0.034). Patients with a maximum LM diameter of ≤ 3.0 cm were associated with a significantly better PFS than patients with a maximum LM diameter of between 3.0 and 5.0 cm (HR: 0.51, 95.0% CI: 0.28–0.92; *p* = 0.026). Additionally, patients with stable or progressive disease after pre-ablation systemic chemotherapy were associated with a significantly poorer PFS than patients with partial remission (HR: 1.54, 95.0% CI: 1.03–2.30 and HR: 2.79, 95.0% CI: 1.86–4.19, respectively; *p* < 0.01). In the multivariate analysis, primary tumor location, the maximum diameter of the LMs, and the patients’ responses to pre-ablation systemic chemotherapy were confirmed as independent predictors of PFS after adjustment for other potential prognostic factors (HR: 0.55, 95.0% CI: 0.31–0.98, *p* = 0.042; HR: 0.448, 95.0% CI: 0.24–0.85, *p* = 0.035; HR: 2.90, 95.0% CI: 1.93–4.36, *p* < 0.01).

## DISCUSSION

In 1990, Bufill [20] proposed that two genetically distinct forms of CRC exist with demarcation at the splenic flexure. Since then, disparities between left- and right-sided CRCs have been investigated in several studies [21, 22]. Different origins (embryonic midgut *vs.* hindgut), genetic and molecular alterations (*BRAF* mutations, chromosomal instability, microsatellite instability, and a CpG island methylator phenotype), invasion subtypes (mucinous *vs.* infiltrating histology), epidemiology, prognoses, recurrence patterns, and therapeutic effectiveness all suggest that the tumor biology differs between left- and right-sided CRCs [8, 23, 24].

Different investigations have revealed that primary tumor location may influence the prognosis of patients with mCRC. A recent study [7] enrolled 2,972 mCRC patients and demonstrated that patients with right-sided mCRC had a poorer OS. Loupakis *et al.* [8] reported a favorable prognosis in patients with left-sided mCRC (*n* = 2,027) after first-line chemotherapy with or without bevacizumab. Similar results were also obtained in a further study of 423 mCRC patients [10].

The liver represents the most common site of distant metastases from CRC. In patients with CRLMs, conflicting results have been obtained regarding the prognostic implication of primary tumor location. Adam *et al.* [25] included 840 patients who had undergone resection for CRLMs and identified right-sided colon cancer as a poor prognostic factor for OS. However, Vigano *et al.* [26] reported no prognostic value of primary tumor location. Two studies respectively enrolled 1,471 and 1,004 patients who underwent hepatectomy for CRLMs and both reported a poorer survival for patients with primary rectal cancer [27, 28]. Several other studies [29–31] have reported no significant differences between primary colon and rectal cancer patients who underwent hepatectomy. Except hepatectomy for CRLMs, studies of other liver-directed therapies have also obtained conflicting results. Rectal origin predicted a poor survival in patients who underwent radioembolization in combination with chemotherapy for CRLMs. However, no prognostic value was determined for rectal origin in a study of hepatic arterial infusion and laser-induced interstitial thermotherapy [32–34].

In recent years, ablative therapies have been regarded as a promising modality for CRLMs due to their minimal invasiveness, reproducibility, low complication rates, and applications for those with a relatively poor physical condition [35]. In a study by Kennedy *et al.* [36], primary rectal cancer was identified as a negative predictor of survival after laparoscopic radiofrequency ablation for CRLMs. Other studies conducted by Agcaoglu *et al.* [37] and Gillams *et al.* [38] reported no predictive value of primary tumor location (left- or right-sided colon *vs.* rectum) on the prognosis of patients (*n* = 395 and *n* = 309, respectively) who underwent radiofrequency ablation for CRLMs.

Compared with radiofrequency ablation, the prognostic value of primary tumor location on patient survival after MWA remains poorly understood. In the present study, we stratified our cohort into three groups (the left-sided colon, the right-sided colon, and the rectum group) according to primary tumor location. The OS outcomes were comparable between the groups, with median values of 35, 33, and 32 months for primary tumors located in the left-sided colon, the right-sided colon, and the rectum, respectively. With regards to PFS outcomes, a better PFS outcome was observed in patients with left-sided colon cancer (median PFS: 17 months) than in patients with right-sided colon cancer (median PFS: 13 months), while no differences were observed between patients with left- or right-sided colon and rectal cancer. These findings are comparable to those of a previous study [25] that demonstrated the inferior prognosis of patients with right-sided primary colon cancer compared to patients with left-sided primary colon cancer after hepatectomy for CRLMs. We hypothesized that there may be several explanations for this difference between left-sided and right-sided CRC. Firstly, the heterogeneous tumor biology of primary CRC may influence the prognosis of patients with CRLMs after MWA. Secondly, the different blood drainage of left-sided CRC and right-sided CRC may partially contribute to the different prognosis after MWA. The splenic vein drains flow from the spleen flexure, descending colon, and the rectum, and the superior mesenteric vein drains flow from the transverse and ascending colon. This is referred to as the “streaming” effect of the portal vein [39, 40], which has been shown to impact on the distribution of LMs from CRC [39, 41]. Thus, we hypothesized that these two explanations may work synergistically to influence the prognosis of patients with CRLMs. However, the definitive explanation still requires investigation. Recently, two studies regarding the clinicopathological characteristics and molecular features of CRC [2, 23] have indicated a gradual change along the colorectal duct rather than an abrupt change at the dividing line, which has challenged the conventional hypothesis of two genetically distinct forms of CRC. Although our study could not definitively answer all the above questions, we have provided new evidence on the differences between CRLM patients based on primary tumor location.

In this study, the maximum diameter of the LMs was found to be an independent prognostic factor for both OS and PFS in the univariate and multivariate analyses. Additionally, the number of CRLMs was also shown to influence PFS in the univariate analysis. This is consistent with prior studies of surgical and ablative approaches [37, 42], which have demonstrated the predictive value of the number and size of CRLMs. Furthermore, the patients’ responses to pre-ablation systemic chemotherapy have also been identified as a predictor of both OS and PFS. These findings are consistent with a study conducted by Stang et al. [43], which reported on the prognostic value of patients’ responses to pre-ablation systemic chemotherapy for CRLMs. In this study, 2.0% of patients reported post-procedural complications, which is comparable with the findings of our previous report (2.6% of 1,136 patients after MWA) [16] and a study conducted by Livraghi et al. (2.9% of 736 patients after MWA) [44].

This study has several limitations. Firstly, the retrospective design of this study may have resulted in selection bias among the groups. Secondly, this study is limited to a single-institutional review with a median follow-up of 24 months. Thirdly, owing to incomplete data, genetic information could not be compared between the groups. Hence, randomized, controlled trials with complete data and longer follow-up periods will need to be conducted.

In conclusion, patients with LMs originating from left-sided CRC presented with better PFS outcomes compared to patients with primary right-sided CRC. Stratification based on primary tumor location should be taken into consideration in the assessment of disease progression in patients who intend to undergo MWA for CRLMs.

## MATERIALS AND METHODS

### Ethical statement

This retrospective study was approved by the Institutional Review Board of the Chinese PLA General Hospital. All ablative procedures were performed in accordance with the Practice Guidelines for Ultrasound-Guided Percutaneous Microwave Ablation for Hepatic Malignancy [45]. Research was conducted in accordance with the Declaration of Helsinki and written informed consent was obtained from each participant prior to ablation.

### Patients and data collection

A prospectively maintained database of patients who underwent percutaneous MWA for CRLMs at our institution between March 2006 and March 2016 was reviewed. The inclusion criteria for this study included: (1) patients who had undergone resection for CRC and had been pathologically diagnosed as having colorectal adenocarcinoma; (2) biopsy confirmed LMs; (3) contraindication to liver surgery due to tumor size and location, poor liver function, advanced-stage disease, medical comorbidities, or patient refusal to undergo hepatectomy; (4) ≤ 3 LMs with a maximum diameter of 5.0 cm (if solitary) or 3.0 cm (if multiple); (5) an absence of portal vein thrombosis or extrahepatic metastases; and (6) the patients’ general condition permits MWA. All of the patients in this study underwent abdominal magnetic resonance imaging or computed tomography prior to liver biopsy. To obtain more accurate conclusions, patients who did not undergo a liver biopsy at our institution were excluded. In addition, patients with ascites, poor general health, a prothrombin time of > 40 seconds, liver failure, contraindications to intravenous anesthesia, who had received other liver-directed treatment prior to ablation, or who were lost to follow-up were also excluded. Pre-operative demographic data, tumor characteristics, and details of the systemic chemotherapy regimens of each patient were recorded. The study cohort was divided into three groups according to the location of the primary tumor: the left-sided colon, the right-sided colon, and the rectum. The demarcation point of the left- and right-sided colon was the splenic flexure [2].

### MWA procedures

Two experienced interventional radiologists (XLY and PL, each with 22 years of experience) performed MWA. Ultrasound-guided percutaneous MWA was performed using a cooled-shaft microwave system (KY-2000, Kangyou Medical, Nanjing, China), which produces a maximum output of 100.0 watts at a frequency of 2,450.0 MHz. MWA procedures were performed under the guidance of conventional ultrasound. If the image was indiscernible from conventional ultrasound, contrast-enhanced ultrasound-guidance (SonoVue^®^; Bracco Imaging, Milan, Italy) was administered as described previously [46]. Twenty-gauge thermocouple needles were inserted into the designated location to monitor the real-time temperature throughout the procedure. After intravenous anesthesia with propofol (6.0–12.0 mg/kg/h) and ketamine (1.0–2.0 mg/kg) by an anesthesiologist, antennas were percutaneously inserted into the tumor under ultrasound guidance. For lesions with a maximum diameter of < 1.7 cm, one antenna was used, and for lesions with a maximum diameter of ≥ 1.7 cm, two antennas were used. Microwave emission did not stop until the temperature measured at the designated location remained at 50.0–54.0°C for ≥ 3 minutes, or had reached 60.0°C [45]. The needle track was cauterized during withdrawal of the antennas to avoid bleeding and tumor seeding. Contrast-enhanced ultrasound was performed immediately after MWA to assess whether tumor inactivation was complete with the hyperechoic area covering the entire tumor with a safety-margin of 5.0–10.0 mm. If tumor inactivation was incomplete, additional MWA sessions were performed.

### Follow-up tests

Procedure-related complications and side-effects were recorded according to the standardized terminology and reporting criteria for image-guided tumor ablation proposed by Ahmed *et al.* [47]. Follow-up tests included contrast-enhanced magnetic resonance imaging, contrast-enhanced ultrasound, and blood examinations (e.g., routine blood, liver function, and tumor biomarker tests), which were performed 1, 3, 6, 9, and 12 months after MWA and, thereafter, at an interval of 3–6 months. Technical success was defined as no contrast enhancement of ablated tumors on contrast-enhanced ultrasound 1 month after MWA treatment. Tumor progression was defined as local recurrence (i.e., contrast enhancement inside or abutting the ablated tumor), intrahepatic metastases in the remnant liver, or extrahepatic metastases. PFS and OS were defined as the interval from the initial MWA until tumor progression or death. Censored cases were defined as having no event until last follow-up.

### Statistical analyses

Quantitative parameters and categorical variables were compared using a one-way analysis of variance, Nemenyi, or Chi-square test as appropriate. PFS and OS curves were plotted using the Kaplan-Meier method. Survival outcomes were stratified according to primary tumor location and compared using the log-rank test. The Cox proportional hazards model was applied for univariate and multivariate analyses of the following factors: age; sex; primary tumor location; tumor-node-metastasis stage; the timing, number, maximum diameter, and distribution of LMs; Karnofsky performance status; histological grade; pre-ablation carcinoembryonic antigen levels; smoking status; and alcohol consumption. All statistical analyses were conducted using Statistical Package for the Social Sciences for Windows, software version 19.0 (SPSS Inc., Chicago, IL, USA). Survival curves were generated using GraphPad Prism for Windows, software version 5.0 (GraphPad Software, Inc., La Jolla, CA, USA). A two-tailed *p* < 0.05 was considered statistically significant.

## Abbreviations

CI: confidence interval
CRC: colorectal cancer
CRLM: colorectal liver metastasis
HR: hazard ratio
LM: liver metastasis
mCRC: metastatic colorectal cancer
MWA: microwave ablation
OS: overall survival
PFS: progression-free survival
